# Modulation of the Nur77-Bcl-2 apoptotic pathway by p38α MAPK

**DOI:** 10.18632/oncotarget.19227

**Published:** 2017-07-13

**Authors:** Jie Liu, Guang-Hui Wang, Ying-Hui Duan, Yi Dai, Yuzhou Bao, Mengjie Hu, Yu-Qi Zhou, Mingyu Li, Fuquan Jiang, Hu Zhou, Xin-Sheng Yao, Xiao-Kun Zhang

**Affiliations:** ^1^ School of Pharmaceutical Sciences, Fujian Provincial Key Laboratory of Innovative Drug Target Research, Xiamen University, Xiamen, China; ^2^ Institutes of Traditional Chinese Medicine and Natural Products, Jinan University, Guangzhou, China; ^3^ Sanford Burnham Prebys Medical Discovery Institute, La Jolla, California, USA

**Keywords:** CCE9, Nur77, Bcl-2, p38α MAPK, apoptosis

## Abstract

Orphan nuclear receptor Nur77 promotes apoptosis by targeting mitochondria through interaction with Bcl-2, an event that converts Bcl-2 from a survival to killer. However, how the Nur77-Bcl-2 apoptotic pathway is regulated remains largely unknown. In this study, we examined the regulation of the Nur77-Bcl-2 pathway by CCE9, a xanthone compound. Our results demonstrated that the apoptotic effect of CCE9 depended on its induction of Nur77 expression, cytoplasmic localization, and mitochondrial targeting. The activation of the Nur77-Bcl-2 pathway by CCE9 was associated with its activation of p38α MAPK. Inhibition of p38α MAPK activation by knocking down or knocking out p38α MAPK impaired the effect of CCE9 on inducing apoptosis and the expression and cytoplasmic localization of Nur77. In addition, CCE9 activation of p38α MAPK resulted in Bcl-2 phosphorylation and Bcl-2 interaction with Nur77, whereas inhibition of p38α MAPK activation or expression suppressed the interaction. Moreover, mutating Ser87 and Thr56 in the loop of Bcl-2, which are known to be phosphorylated by p38α MAPK, impaired the ability Bcl-2 to interact with Nur77. Together, our results reveal a profound role of p38α MAPK in regulating the Nur77-Bcl-2 apoptotic pathway through its modulation of Nur77 expression, Bcl-2 phosphorylation, and their interaction.

## INTRODUCTION

Nur77 (also called TR3, NGFI-B, or NR4A1), an early immediate-response gene, is perhaps the most potent pro-apoptotic member of the nuclear receptor superfamily [1–4]. Besides being a nuclear transcriptional factor, Nur77 acts nongenomically in the cytoplasm by targeting mitochondria to induce apoptosis in response to certain apoptotic stimuli [5–15]. Mitochondrial targeting of Nur77 involves its interaction with Bcl-2 [7, 13, 15–18]. The interaction results in a Bcl-2 conformational change that converts Bcl-2 from an anti-apoptotic to a pro-apoptotic molecule [7, 13, 15–18]. Interestingly, the Nur77-Bcl-2 interaction is mediated by an unexpected protein-protein interaction site in the natively unstructured loop of Bcl-2 located between BH3 and BH4 domains [7, 15, 18], which has been shown to play a critical role in regulating Bcl-2 function [19, 20]. However, how the binding of Nur77 to the loop of Bcl-2 is regulated remains undefined.

Previous reports indicate that phosphorylation of Bcl-2 is an important mechanism regulating the anti-apoptotic effect of Bcl-2 [19, 20]. Phosphorylation of Bcl-2 is mainly mediated by multiple phosphorylation sites in its loop region [19, 20]. Among kinases involved in Bcl-2 loop phosphorylation, p38α MAPK that regulates a variety of cellular responses to environmental stress, pro-inflammatory cytokines, lipopolysaccharide (LPS) and other signals [21, 22] has been shown to phosphorylate Ser87 and Thr56 located within the loop of Bcl-2 [23, 24]. Phosphorylation of Bcl-2 abrogates its anti-apoptotic function in response to nerve growth factor (NGF) withdrawal or treatment with tumor necrosis factor (TNF), paclitaxel, arsenic trioxide, and NO [24, 25]. Under these conditions, Bcl-2 phosphorylation by p38α MAPK leads to pro-apoptotic events [24]. Interestingly, apoptosis provoked by several stimuli that activate p38α MAPK is closely linked to Bcl-2-expressing populations [23, 24], demonstrating the death potential of the Bcl-2 protein upon p38α MAPK phosphorylation.

Xanthones, a class of three-membered heterocyclic ring compounds mainly found as secondary metabolites in higher plants and microorganisms, exert diverse biological activities, including anti-hypertensive, anti-oxidative, anti-thrombotic, and anti-cancer activity, depending on their structure features [26, 27]. A large number of naturally occurring and synthetic xanthones, such as psorospermin, dimethylxanthesone-4-acetic acid (DMXAA) and α-mangostin, exert potent anti-cancer activities in a variety of cancer cells, including breast cancer, colorectal cancer, hepatoma, leukemia, and small cell lung cancer [26–29]. We reported here that 1,3,7-trihydroxy-2,4-diprenylxanthone (named CCE9) isolated from the Chinese medicinal plant, *Cratoxylum formosum ssp. Pruniflorum* [30] is a positive regulator of the Nur77-Bcl-2-dependent apoptotic pathway. Our results demonstrated that CCE9 could induce both Nur77 expression and Bcl-2 phosphorylation in a p38α MAPK dependent manner, resulting in Nur77 interaction with Bcl-2 and Nur77 cytoplasmic localization. Furthermore, we showed that p38α MAPK phosphorylation of Ser87 and Thr56 in the loop of Bcl-2 was essential for its interaction with Nur77. Our results therefore reveal a critical role of p38α MAPK in the regulation of the Nur77-Bcl-2 apoptotic pathway.

## RESULTS

### CCE9 induces apoptosis in a Nur77 and Bcl-2 dependent manner

**Figure 1:**
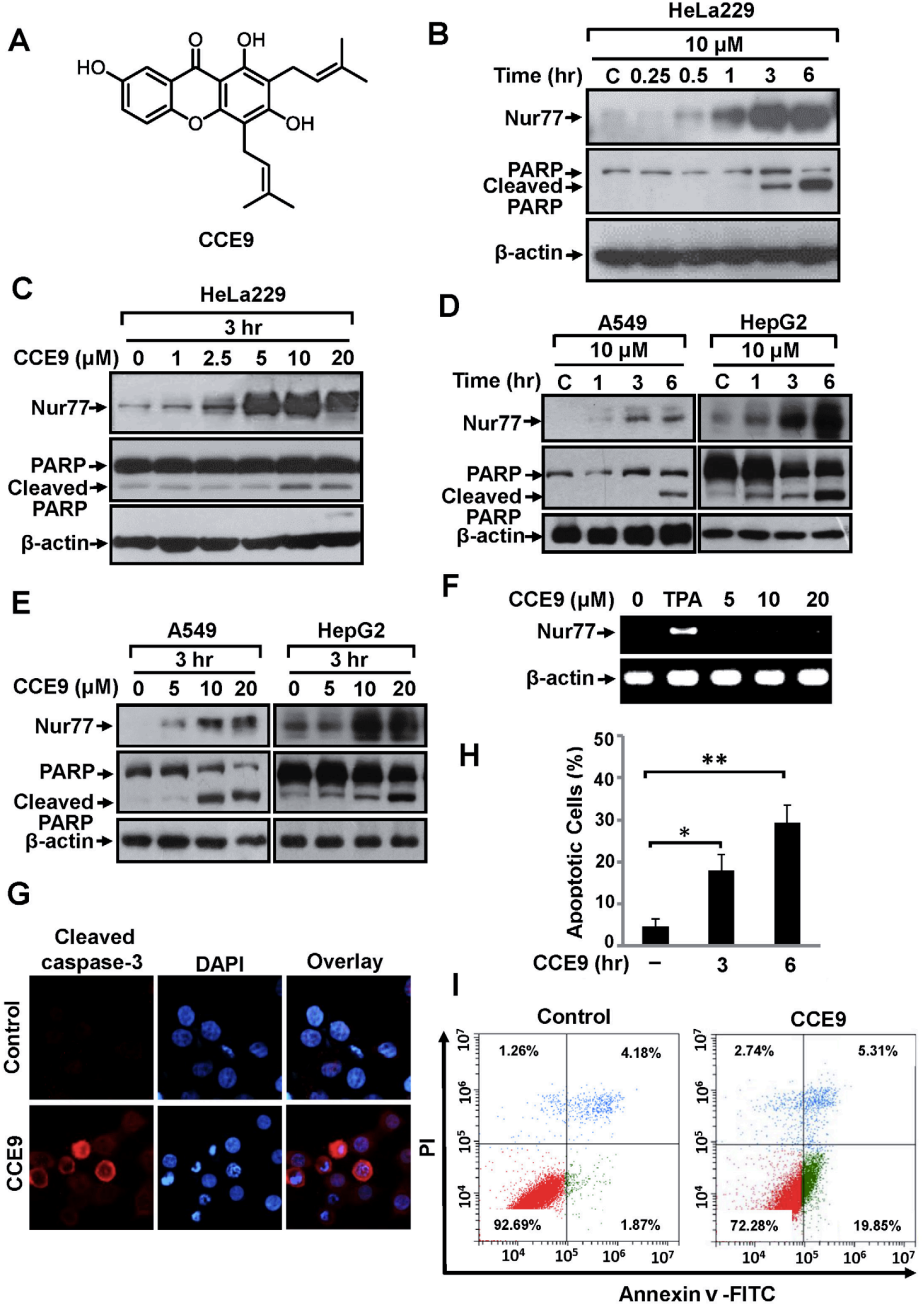
CCE9 induces Nur77 expression and apoptosis **(A)** Structure of CCE9. **(B)** Time-course analysis of Nur77 and PARP cleavage induction by CCE9. HeLa229 cells treated with 10 μM CCE9 for the indicated time were determined by Western blotting using anti-Nur77 antibody. **(C)** Dose dependent effect of CCE9. HeLa229 cells treated with vehicle or indicated concentration of CCE9 for 3 hr were analyzed for Nur77 expression and PARP cleavage by Western blotting. **(D)** Time-course analysis of Nur77 expression and apoptosis induction by CCE9 in A549 and HepG2 cells. Cells treated with 10 μM CCE9 for the indicated time were analyzed for Nur77 expression and PARP cleavage by Western blotting. **(E)** Dose-dependent induction of Nur77 and apoptosis by CCE9 in A549 and HepG2 cells. Cells treated with vehicle or the indicated concentration of CCE9 for 3 hr were analyzed for Nur77 expression and PARP cleavage by Western blotting. **(F)** RT-PCR analysis of Nur77 mRNA expression in HeLa229 cells. Cells treated with vehicle, TPA (100 ng/ml), or indicated concentration of CCE9 for 3 hr. Nur77 and β-actin mRNA products were simultaneously amplified in the same reaction system, in which β-actin expression level served as an internal control. **(G)** Caspase-3 activation by CCE9. HeLa229 cells were treated with 10 μM CCE9 for 3 hr, immunostained with antibody recognizing the cleaved caspase-3. Nuclei were visualized by co-staining with DAPI. **(H)** DAPI staining. HeLa229 cells were treated with CCE9 (10 μM) or vehicle for 3 hr or 6 hr and subjected to DAPI staining. Apoptotic cells were scored and compared between different treatments. *, P<0.01 (VS. control); **, P<0.01 (VS. control). **(I)** The apoptotic effect of CCE9. HeLa229 cells were treated with vehicle or 10 μM CCE9 for 6 hr and stained with Annexin V/PI. Apoptosis was analyzed by fluorescence-activated cell sorting analysis.

To identify new modulators of the Nur77-Bcl-2 apoptotic pathway, we screened a natural product library prepared from Chinese herbal medicines, and found that CCE9 (Figure 1A) could potently increase Nur77 expression and apoptosis. In HeLa229 cells, CCE9 induced a rapid increase of Nur77 expression with maximum induction in cells treated with CCE9 for 3 hr and 6 hr (Figure 1B). CCE9 also showed a dose-dependent induction of Nur77 expression. Although CCE9 induction of Nur77 could be seen at 1 μM concentration, significant Nur77 induction was observed when 5 μM or higher dose of CCE9 was used (Figure 1C). Levels of Nur77 protein in A549 and HepG2 cells were also induced by CCE9 in a time- (Figure 1D) and dose-dependent (Figure 1E) manner. We also determined whether CCE9 could induce Nur77 mRNA expression. HeLa229 cells treated with vehicle or with CCE9 at 5, 10, 20 μM for 3 hr were examined for levels of Nur77 transcript by reverse transcription-PCR (RT-PCR). While strong induction of Nur77 mRNA expression was seen when cells were treated with phorbol-12-myristate-13-acetate (TPA), no apparent induction of Nur77 mRNA level by CCE9 was found (Figure 1F). Thus, the induction of Nur77 protein by CCE9 was not due to its transcriptional regulation of Nur77 expression.

The death effect of CCE9 was examined by assessing its ability to induce PARP cleavage [5]. Our data showed that PARP was cleaved by CCE9 treatment, which correlated well with its induction of Nur77 expression (Figure 1B, 1C). CCE9 also induced both Nur77 expression and PARP cleavage in A549 cells and HepG2 cells in a time- and dose-dependent manner (Figure 1D, 1E). The apoptotic effect of CCE9 was also illustrated by its activation of caspase-3 in HeLa229 cells revealed by immunostaining (Figure 1G). In addition, DAPI staining showed that treatment of HeLa229 cells by CCE9 for 3 hr resulted in about 18.0% of cells displaying nuclear condensation and fragmentation, which was increased to 29.3% when cells were treated with CCE9 for 6 hr (Figure 1H). Furthermore, Annexin V/PI staining showed about 19.85% HeLa229 cells undergoing extensive early apoptosis (Annexin V^+^/PI^-^) when they were treated with 10 μM CCE9 for 6 hr (Figure 1I).

**Figure 2:**
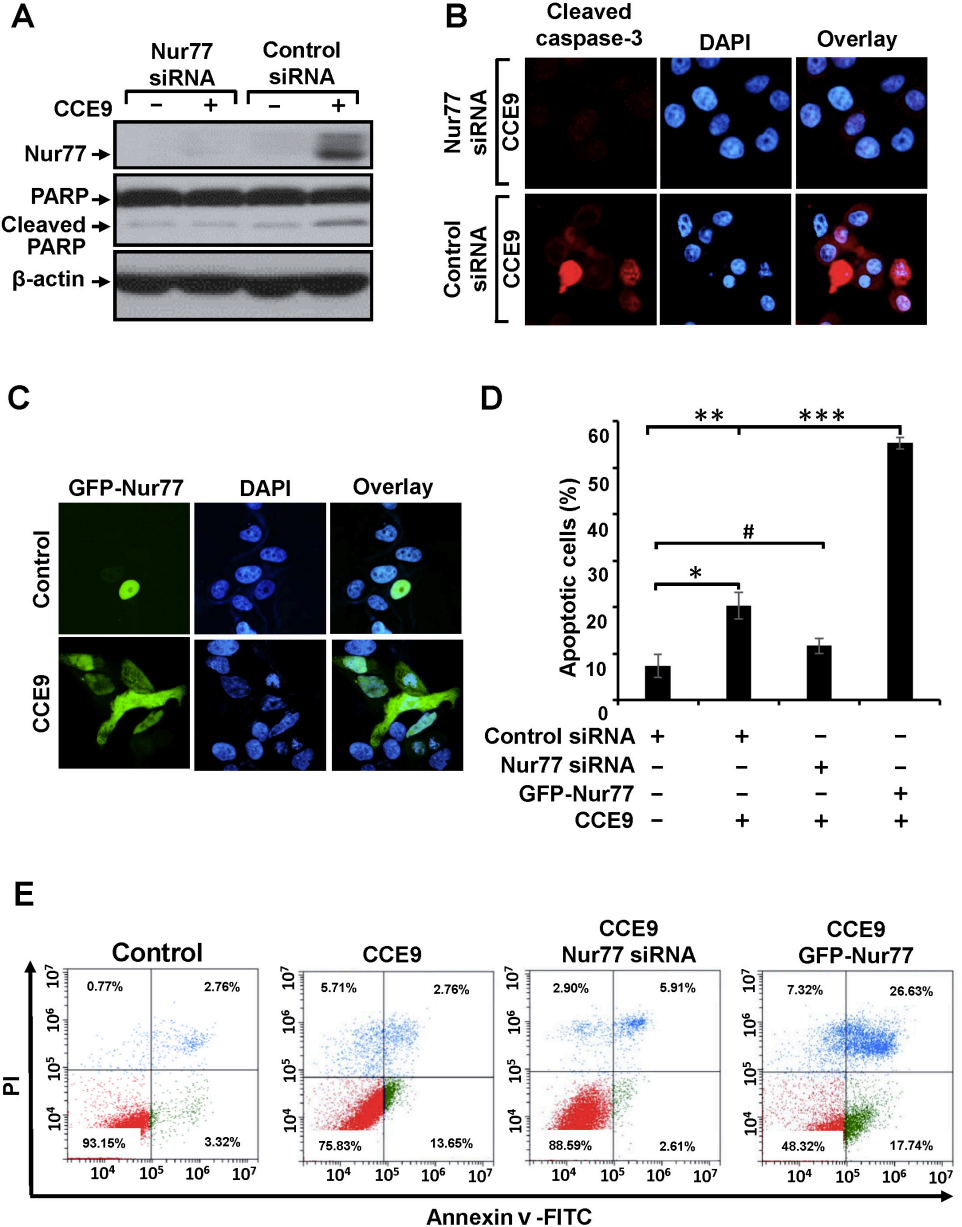
Role of Nur77 in CCE9 induced apoptosis **(A-B)** Transfection of Nur77 siRNA inhibits apoptosis induction by CCE9. HeLa229 cells transfected with control or Nur77 siRNA were treated with CCE9 (10 μM) for 3 hr, and analyzed for PARP cleavage by Western blotting **(A)**, caspase-3 activation by immunostaining **(B)**. **(C)** Transfection of Nur77 promotes apoptosis induction by CCE9. HeLa229 cells transfected with GFP-Nur77 expression vector were treated with 10 μM of CCE9 for 3 hr. Subcellular localization of GFP-Nur77 was examined by confocal microscopy. **(D)** Nur77 mediates the apoptotic effect of CCE9. HeLa229 cells were transfected with Nur77 siRNA and GFP-Nur77and subjected to CCE9 treatment (10 μM) or vehicle in serum-free medium for 3 hr. Apoptotic cells examined by DAPI staining were compared between different cells. *, P < 0.01 (vs. control); **, P < 0.01 (vs. control); ***, P < 0.01 (vs. control siRNA with CCE9 treatment); #, P>0.05 (vs. control). **(E)** The apoptotic effect of CCE9 is impaired in Nur77 knockdown and Nur77 overexpressed cells. HeLa229 cells transfected with Nur77 siRNA and HeLa229 cells transfected with GFP-Nur77 were treated with vehicle or 10 μM of CCE9 for 6 hr and stained with Annexin V/PI. Apoptosis was analyzed by fluorescence-activated cell sorting analysis.

Our observation that CCE9-induced apoptosis was accompanied with its induction of Nur77 expression promoted us to determine whether Nur77 expression plays a role in CCE9 induction of apoptosis. Thus, Nur77 siRNA was transfected into HeLa229 cells, which almost completely suppressed the induction of Nur77 expression by CCE9 (Figure 2A). Inhibition of Nur77 expression by Nur77 siRNA transfection impaired the ability of CCE9 to induce PARP cleavage (Figure 2A). Caspase-3 activation by CCE9 was also reduced by transfection of Nur77 siRNA (Figure 2B). We also used DAPI staining to evaluate the effect of Nur77 expression. CCE9 treatment resulted in about 20.3% cells displaying apoptotic nuclear morphology such as nuclear condensation and fragmentation when cells were transfected with control siRNA, which was reduced to 11.7% when cells were transfected with Nur77 siRNA (Figure 2D). We also examined whether overexpression of Nur77 could enhance the death effect of CCE9. HeLa229 cells were transfected with GFP-Nur77 (Figure 2C) and subsequently treated with CCE9. About 55.3% GFP-Nur77-transfected cells displayed nuclear condensation and fragmentation (Figure 2D). To further determine the role of Nur77 expression, HeLa229 cells transfected with either Nur77 siRNA or GFP-Nur77 were evaluated by Annexin V/PI staining. Transfection of Nur77 siRNA inhibited the death effect of CCE9 on inducing early apoptosis, revealed by the reduction of the percentage of Annexin V^+^/PI^-^ cells from 13.65% to 2.61% (Figure 2E). In contrast, transfection of GFP-Nur77 enhanced the apoptotic effect of CCE9, as its induction of late apoptosis (Annexin V^+^/PI^+^ cells) was increased from 2.76% to 26.63%. Taken together, these studies demonstrated that Nur77 plays a critical role in mediating the apoptotic effect of CCE9.

**Figure 3:**
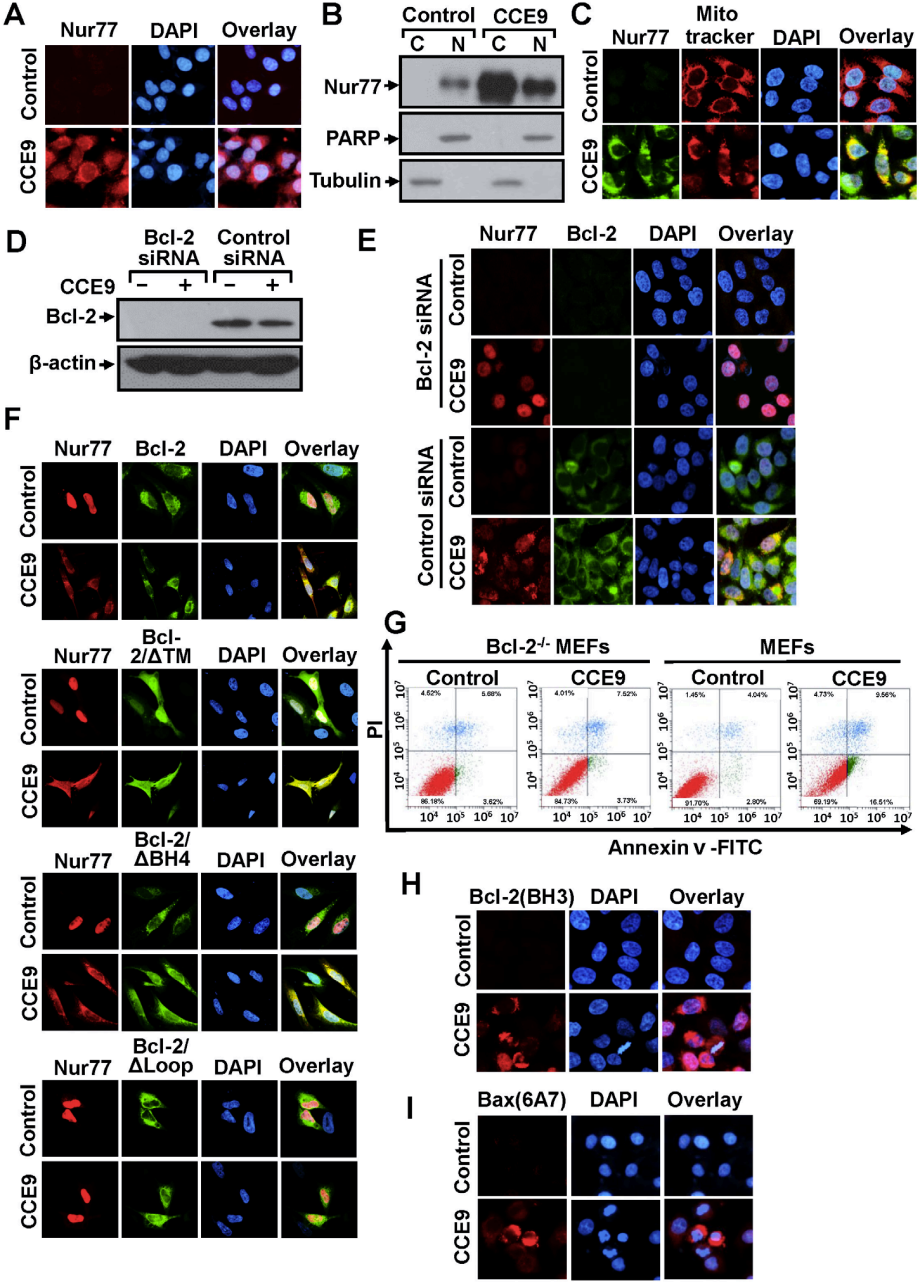
CCE9 induces Nur77 mitochondrial targeting, Bcl-2 colocalization, and Bcl-2 conformational change **(A)** CCE9 induces cytoplasmic localization of Nur77. HeLa229 cells treated with 10 μM CCE9 for 2 hr were subjected to immunostaining with anti-Nur77 antibody. Cells were co-stained with DAPI to visualize the nuclei. **(B)** Cellular fractionation. Nuclear and cytoplasmic fractions were prepared from HeLa229 cells treated with 10 μM CCE9 for 2 hr, subjected to Western blotting analysis. The purity of cellular fraction was confirmed by using anti-PARP and anti-Tubulin antibodies. **(C)** Nur77 mitochondrial targeting. HeLa229 cells treated with 10 μM CCE9 for 2 hr, Nur77 expression was immunostained with anti-Nur77 antibody and analyzed by confocal microscopy. Cells were co-stained with Mitotraker to recognize the mitochondria and with DAPI to visualize the nuclei. **(D-E)** The effect of Bcl-2 siRNA transfection on Nur77 cytoplasmic localization. HeLa229 cells transfected with Bcl-2 siRNA or control siRNA for 48 hr and subsequently treated with CCE9 for 2 hr were examined for Bcl-2 expression by Western blotting **(D)** or subcellular localization of Nur77 by immunostaining **(E)** Cells were co-stained with DAPI to visualize the nuclei. **(F)** Co-localization of Nur77 with Bcl-2 mutants. Bcl-2-depleted HeLa cells were transfected with Nur77 and the indicated Bcl-2 or mutants for 24 hr and subsequently treated with CCE9 for 3 hr. Subcellular localization of Nur77 and Bcl-2 was examined by immunostaining. **(G)** The apoptotic effect of CCE9 is impaired in Bcl-2 knockout MEFs. MEFs or Bcl-2^-/-^ MEFs were treated with vehicle or 10 μM CCE9 for 6 hr and stained with Annexin V/PI. Apoptosis was analyzed by fluorescence-activated cell sorting analysis. **(H)** Bcl-2 conformational change. HeLa229 cells treated with 10 μM of CCE9 for 2 hr were examined for Bcl-2 conformational change by anti–Bcl-2(BH3) antibody. Cells were also stained with DAPI to visualize the nucleus. **(I)** Bax activation. HeLa229 cells treated with 10 μM of CCE9 for 2 hr were examined for Bax activation by conformation-sensitive anti-Bax antibody, Bax (6A7). Cells were also stained with DAPI to visualize the nucleus.

Transfected GFP-Nur77 was found exclusively in the nucleus of cells. However, GFP-Nur77 could be detected in both the nucleus and cytoplasm when cells were treated with CCE9 (Figure 2C). This observation suggested that CCE9 might be involved in inducing Nur77 cytoplasmic localization, the hallmark of the Nur77-Bcl-2 apoptotic pathway [7]. To confirm the effect of CCE9 on inducing the cytoplasmic localization of Nur77, we studied the subcellular localization of endogenous Nur77 in the presence or absence of CCE9 by immunostaining. In the absence of treatment, endogenous Nur77 protein was hardly detected. Consistent with its ability to induce Nur77 protein expression, CCE9 treatment resulted in a strong Nur77 staining both in the nucleus and cytoplasm of cells with predominant cytoplasmic staining in some cells (Figure 3A). To confirm the effect of CCE9 on inducing Nur77 cytoplasmic localization, cytosolic and nuclear fractions of cells treated with CCE9 were prepared and analyzed for levels of Nur77 protein by immunoblotting. In control cells, the level of Nur77 protein was low and it was exclusively detected in the nuclear fraction. When cells were treated with CCE9, the level of Nur77 protein was dramatically increased especially in the cytoplasmic fraction (Figure 3B). These results demonstrated that CCE9-induced Nur77 is predominantly cytoplasmic. To determine whether CCE9-induced Nur77 targeted at mitochondria, cells treated with CCE9 were stained with anti-Nur77 antibody and Mitotracker, a mitochondrial-selective fluorescent label commonly used in confocal microscopy and flow cytometry [5]. Confocal microscopy showed that the distribution of Nur77 overlapped extensively with that of Mitotracker in cells treated with CCE9 (Figure 3C), suggesting that the cytoplasmic Nur77 induced by CCE9 could target mitochondria. Thus, CCE9 induces not only the expression of Nur77 but also its cytoplasmic localization and mitochondrial targeting.

Cytoplasmic Nur77 is known to bind Bcl-2 [7]. HeLa229 cells transfected with or without Bcl-2 siRNA were then used to determine whether cytoplasmic Nur77 associated with Bcl-2. Transfection of HeLa229 cells with Bcl-2 siRNA almost completely inhibited Bcl-2 expression in the absence or presence of CCE9 (Figure 3D). Immunostaining showed that CCE9 treatment resulted in an extensive colocalization of the cytoplasmic Nur77 with Bcl-2 in cells treated transfected with control siRNA (Figure 3E), suggesting their association in the cytoplasm. However, when cells were transfected with Bcl-2 siRNA, CCE9-induced Nur77 protein was found predominantly in the nucleus (Figure 3E). These results demonstrated that CCE9-induced Nur77 protein associated with Bcl-2 in the cytoplasm, and importantly they suggested that Bcl-2 might act to retain CCE9-induced Nur77 in the cytoplasm likely through their interaction. The loop region of Bcl-2 is required for binding to Nur77 [7]. To determine whether Bcl-2 plays a role in the cellular distribution of Nur77 through their interaction, Bcl-2 mutants with different ability to bind Nur77 were transfected into Bcl-2-depleted HeLa cells to determine their effect of cytoplasmic localization of co-transfected Nur77. Immunostaining showed that transfection of full-length Bcl-2, Bcl-2/Δ TM, and Bcl-2/Δ BH4, which are known to interact with Nur77 [7], could retain co-transfected Nur77 in the cytoplasm. Conversely, co-transfection of Bcl-2/Δ loop, a mutant incapable of binding Nur77 [7], had no such an effect (Figure 3F). These results revealed not only the association of CCE9-induced cytoplasmic Nur77 with Bcl-2 but also a critical role of Bcl-2 expression in retaining Nur77 in the cytoplasm. We next determined whether the expression of Bcl-2 mediated the apoptotic effect of CCE9 using mouse embryonic fibroblasts (MEFs) and Bcl-2 knockout MEFs (Bcl-2^-/-^MEFs). Annexin V/PI staining showed that CCE9 treatment caused a significant early apoptosis in MEFs (Annexin V^+^/PI^-^; 16.51%), while the same treatment hardly induced apoptosis in Bcl-2^-/-^MEFs (Annexin V^+^/PI^-^; 3.73%) (Figure 3G). Thus, the apoptotic effect of CCE9 also depends on Bcl-2 expression.

Nur77 interaction with Bcl-2 results in a Bcl-2 conformational change with its BH3 domain exposed [7]. Such a Bcl-2 conformation is pro-apoptotic and can be detected by antibody against the BH3 domain of Bcl-2, anti-Bcl-2(BH3) [7, 15]. Despite the expression of Bcl-2, HeLa229 cells were not immunostained by the anti-Bcl-2(BH3) antibody, suggesting that Bcl-2 expressed in the cells was anti-apoptotic. In contrast, cells treated with CCE9 exhibited a strong Bcl-2(BH3) immunostaining (Figure 3H), an indication of Bcl-2 conformational change from an anti-apoptotic to a pro-apoptotic molecule [7, 15]. Pro-apoptotic Bcl-2 acts like a BH3-only protein known to activate Bax [8, 15]. Indeed, cells treated with CCE9 were strongly stained by the conformation-sensitive anti-Bax (6A7) antibody that recognizes an N-terminal epitope exposed after Bax activation [31] (Figure 3I), revealing the ability of CCE9 to activate Bax.

### CCE9 induction of apoptosis depends on its activation of p38α MAPK

**Figure 4:**
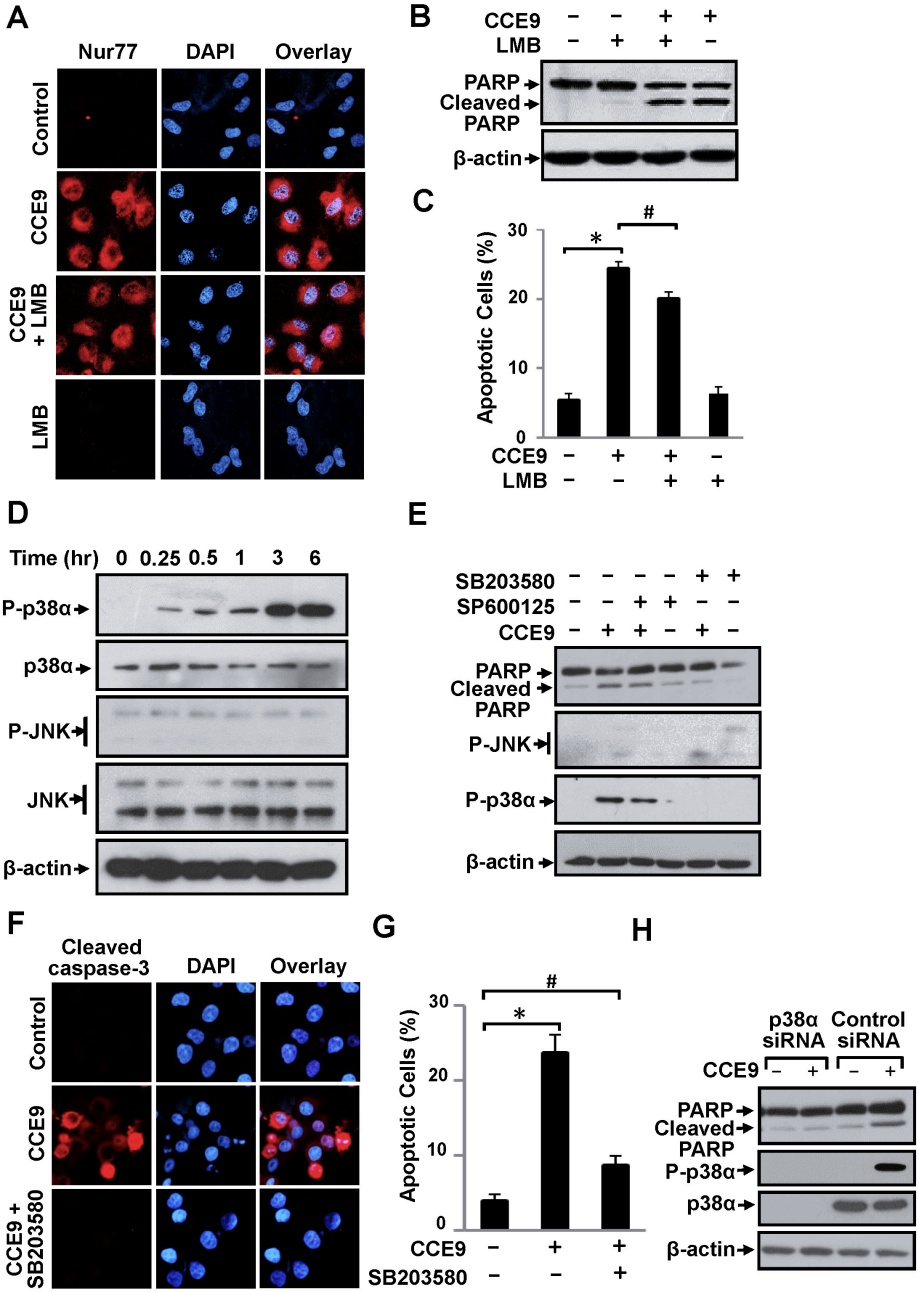
p38α MAPK activation by CCE9 is required for apoptosis and Nur77 expression **(A)** LMB fails to block CCE9-induced Nur77 cytoplasmic localization. HeLa229 cells treated with 10 μM of CCE9 in the presence or absence of LMB (1 ng/mL) for 2 hr were examined by immunostaining using anti-Nur77 antibody. **(B-C)** Effect of LMB on apoptosis induction by CCE9. HeLa229 cells treated with 10 μM CCE9 in the presence or absence of LMB (1 ng/mL) for 3 hr were studied for apoptosis by PARP cleavage by Western blotting **(B)** and DAPI staining, *, P < 0.01 (vs. control); #, P>0.05 (vs. CCE9 treatment) **(C)**. **(D)** Induction of p38 MAPK activation by CCE9. HeLa229 cells treated with 10 μM CCE9 for 0, 0.25, 0.5, 1, 3, 6 hr were analyzed for activation of p38 MAPK and JNK by Western blotting using antibody recognizing phosphorylated (P-) p38 MAPK or JNK. Total p38 MAPK and JNK was used as loading control. **(E)** Role of CCE9 activation of p38 MAPK in PAPR cleavage. HeLa229 cells treated for 3 hr with 10 μM CCE9 in the presence or absence of 25 μM SB203580 or SP600125 were analyzed by Western blotting. **(F-G)** Effect of SB203580 on CCE9 apoptotic activities. HeLa229 cells treated for 3 hr with 10 μM CCE9 in the presence or absence of 25 μM SB203580 were examined by for caspase-3 activation by immunostaining with antibody recognizing the cleaved caspase-3 **(F)** and apoptosis by DAPI staining, *, P < 0.01 (vs. control); #, P>0.05 (vs. control) **(G)**. **(H)** Effect of p38α MAPK siRNA transfection on CCE9 apoptotic activities. HeLa229 cells transfected with p38α MAPK siRNA or control siRNA for 48 hr, and treated with 10 μM CCE9 for 3 hr were analyzed by Western blotting were analyzed by Western blotting.

A variety of protein kinases, including the MAPK family members, are known to regulate the Nur77-Bcl-2 apoptotic pathway [9, 11, 32, 33]. We previously reported that 3-Cl-AHPC activation of the Nur77-Bcl-2 apoptotic pathway involved its induction of CRM-1-dependent Nur77 nuclear export through activation of Jun N-terminal kinase (JNK) [9, 11, 32, 33]. Our current observation that inhibition of Bcl-2 expression prevented CCE9 induction of Nur77 cytoplasmic localization (Figure 3E) suggested that CCE9 might regulate the Nur77-Bcl-2 apoptotic pathway through a different mechanism. Indeed, leptomycin B (LMB), an inhibitor of CRM-1-dependent nuclear export [34], had no effect on CCE9-induced Nur77 cytoplasmic localization (Figure 4A). LMB was also ineffective on the induction of PARP cleavage (Figure 4B) and apoptosis (Figure 4C) by CCE9. Furthermore, JNK activation known to promote Nur77 nuclear export [9, 11, 32, 33] did not occur in HeLa229 cells treated with CCE9 (Figure 4D). Instead, we found that p38α MAPK was rapidly and strongly activated by CCE9 in HeLa229 cells (Figure 4D). To determine the role of CCE9 activation of p38α MAPK, we first used SB203580, a chemical inhibitor known to inhibit both p38α and p38β MAPK. Treatment of HeLa229 cells with SB203580 but not a JNK inhibitor (SP600125) almost completely suppressed the effect of CCE9 on inducing PARP cleavage (Figure 4E). SB203580 also suppressed the effect of CCE9 on activating caspase-3 (Figure 4F) and apoptosis (Figure 4G). We next used p38α-specific siRNA approach to determine the effect of CCE9 on inducing p38 MAPK activation and apoptosis. Transfection of cells with p38α-specific siRNA completely inhibited the expression of p38 MAPK and its activation by CCE9 (Figure 4H). Thus, CCE9 mainly activates p38α isoform but not other isoforms. Similar to the effect of SB203580, transfection of p38α-specific siRNA largely impaired the effect of CCE9 on inducing PARP (Figure 4H). Thus, p38α MAPK activation is essential for the apoptotic effect of CCE9.

### p38α MAPK activation in Nur77 expression, Bcl-2 phosphorylation, and Bcl-2 conformational change

**Figure 5:**
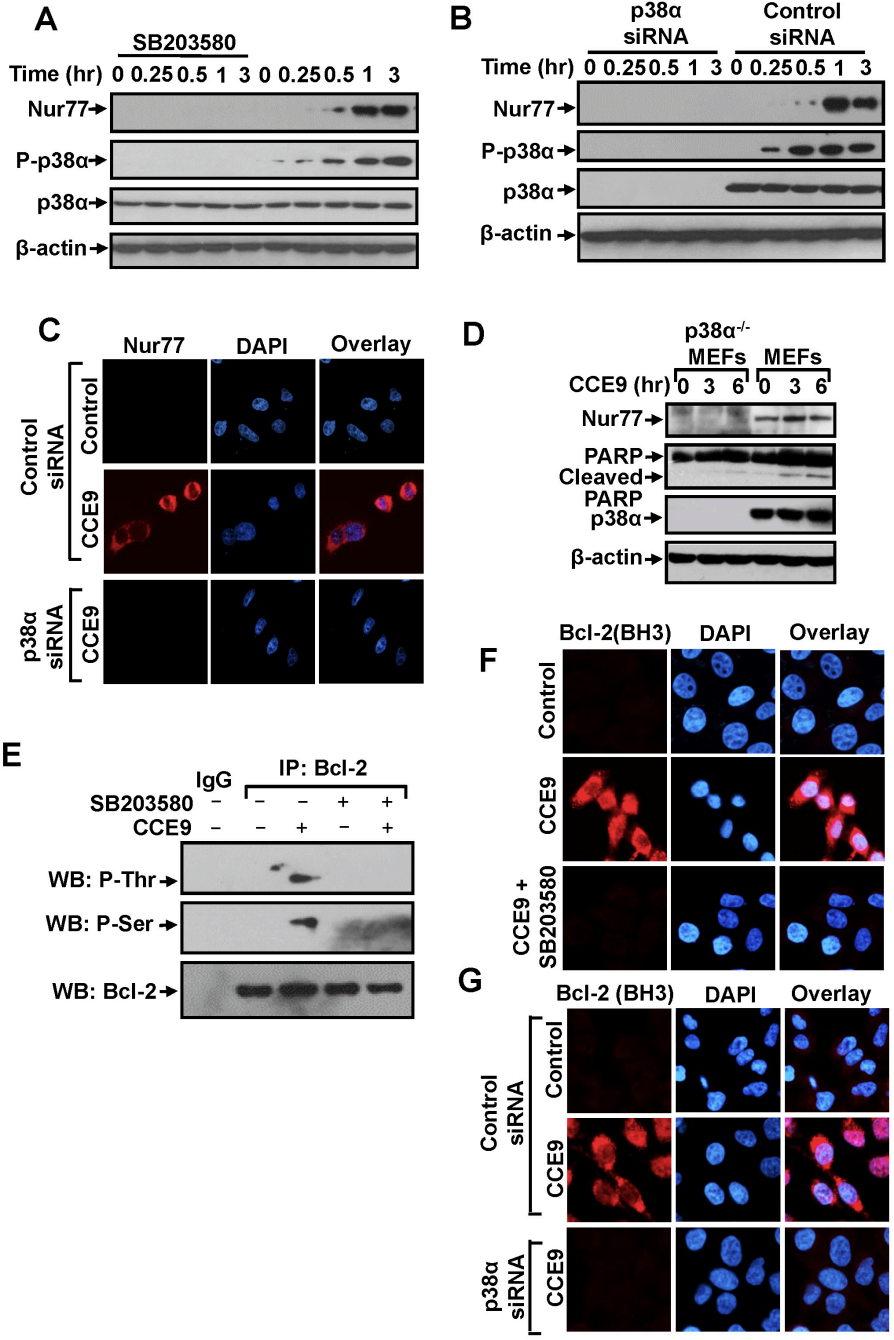
p38α MAPK activation by CCE9 promotes Nur77 expression, Bcl-2 phosphorylation and Bcl-2 conformational change **(A)** Effect of SB203580 on induction of Nur77 expression by CCE9. HeLa229 cells treated with 10 μM CCE9 for 0, 0.25, 0.5, 1, 3 hr in the presence or absence of 25 μM SB203580 were analyzed by Western blotting. **(B-C)** Effect of p38α MAPK siRNA transfection on induction of Nur77 expression by CCE9. HeLa229 cells transfected with p38α MAPK siRNA or control siRNA for 48 hr, and treated with 10 μM CCE9 for the indicated time were analyzed by Western blotting **(B)** and immunostaining **(C)** for Nur77 expression. **(D)** Effect of CCE9 on Nur77 expression and PARP cleavage in MEFs and p38α MAPK^-/-^MEFs. MEFs or p38α MAPK^-/-^MEFs treated with 10 μM CCE9 for 3 hr and 6 hr were analyzed by Western blotting for Nur77 protein expression and p38 MAPK activation. **(E)** CCE9 induced p38α MAPK-dependent Bcl-2 phosphorylation. HeLa229 cells treated for 2 hr with 10 μM CCE9 in the presence or absence of 25 μM SB203580 were subject to immunoprecipitated (IP) by using anti-Bcl-2 antibody. Immunoprecipitates were examined by Western blotting (WB) using anti-phosphorylated (P-) Ser and Thr antibodies. The same membranes were blotted with anti-Bcl-2 antibody to determine immunoprecipitation specificity. **(F)** Effect of SB203580 on CCE9 induced Bcl-2 conformational change. HeLa229 cells treated for 2 hr with 10 μM CCE9 in the presence or absence of 25 μM SB203580, Bcl-2 conformational change by immunostaining with the anti-Bcl-2 (BH3) antibody. **(G)** Effect of p38α MAPK siRNA transfection on CCE9 induced Bcl-2 conformational change. HeLa229 cells transfected with p38α MAPK siRNA or control siRNA for 48 hr, and treated with 10 μM CCE9 for 2 hr, Bcl-2 conformational change by immunostaining with the anti-Bcl-2 (BH3) antibody.

Nur77 expression is required for the apoptotic effect of CCE9. The observation that p38α MAPK inhibitor effectively suppressed CCE9-induced apoptosis suggested that p38α MAPK might modulate the Nur7 apoptotic pathway. We first studied the effect of p38α MAPK on CCE9 induction of Nur77 expression. Treatment of HeLa229 cells with the p38α MAPK inhibitor SB203580 completely suppressed the dose-dependent induction of Nur77 expression (Figure 5A). Knocking down p38α MAPK mRNA expression by transfecting p38α MAPK siRNA also abolished the effect of CCE9 on inducing Nur77 expression and cytoplasmic localization (Figure 5B-5C). We also used p38α knockout MEFs, p38α^-/-^MEFs, to study the role of p38α activation. Treatment of MEFs with CCE9 resulted in induction of Nur77 expression and PARP cleavage. In contrast, CCE9 had little effect on Nur77 expression and PARP cleavage in p38α^-/-^MEFs (Figure 5D). Thus, CCE9 induction of Nur77 expression requires its activation of p38α MAPK.

p38α MAPK has been shown to phosphorylate Bcl-2 and regulate its survival activity [23–25]. To determine whether and how CCE9 activation of p38α MAPK regulated the Nur77-Bcl-2 apoptotic pathway, we studied the possibility that p38α MAPK regulated Nur77-dependent apoptosis by phosphorylating Bcl-2. Bcl-2 was immunoprecipitated with anti-Bcl-2 antibody from HeLa229 cells treated with or without CCE9, and the immunoprecipitated Bcl-2 was then analyzed for phosphorylation by anti-phosphor-Ser and anti-phosphor-Thr antibodies. Our results showed that Bcl-2 immunoprecipitated from CCE9-treated cells reacted strongly with both anti-phospho-Ser and anti-phospho-Thr antibodies, while Bcl-2 immunoprecipitates from non-treated cells did not. Moreover, Bcl-2 immunorecipitates from cells treated with CCE9 in the presence of SB203580 failed to react with anti-phospho-Ser and anti-phospho-Thr antibodies (Figure 5E). These results are consistent with previous report that p38α MAPK could phosphorylate Bcl-2 [23] and suggest that CCE9 is capable of inducing p38α MAPK dependent phosphorylation of Bcl-2. Conversion of Bcl-2 from an anti-apoptotic to a pro-apoptotic molecule is characterized by the exposure of its BH3 domain [7]. Immunostaining showed that the apoptotic effect of CCE9 on inducing BH3 domain staining was strongly attenuated in cells treated with the p38α MAPK inhibitor SB203580 (Figure 5F) or transfected with p38α MAPK siRNA (Figure 5G). Thus, p38α MAPK activation by CCE9 plays a critical role in its induction of Bcl-2 conversion into a death factor.

### Regulation of Nur77-Bcl-2 interaction by p38α MAPK activation

**Figure 6:**
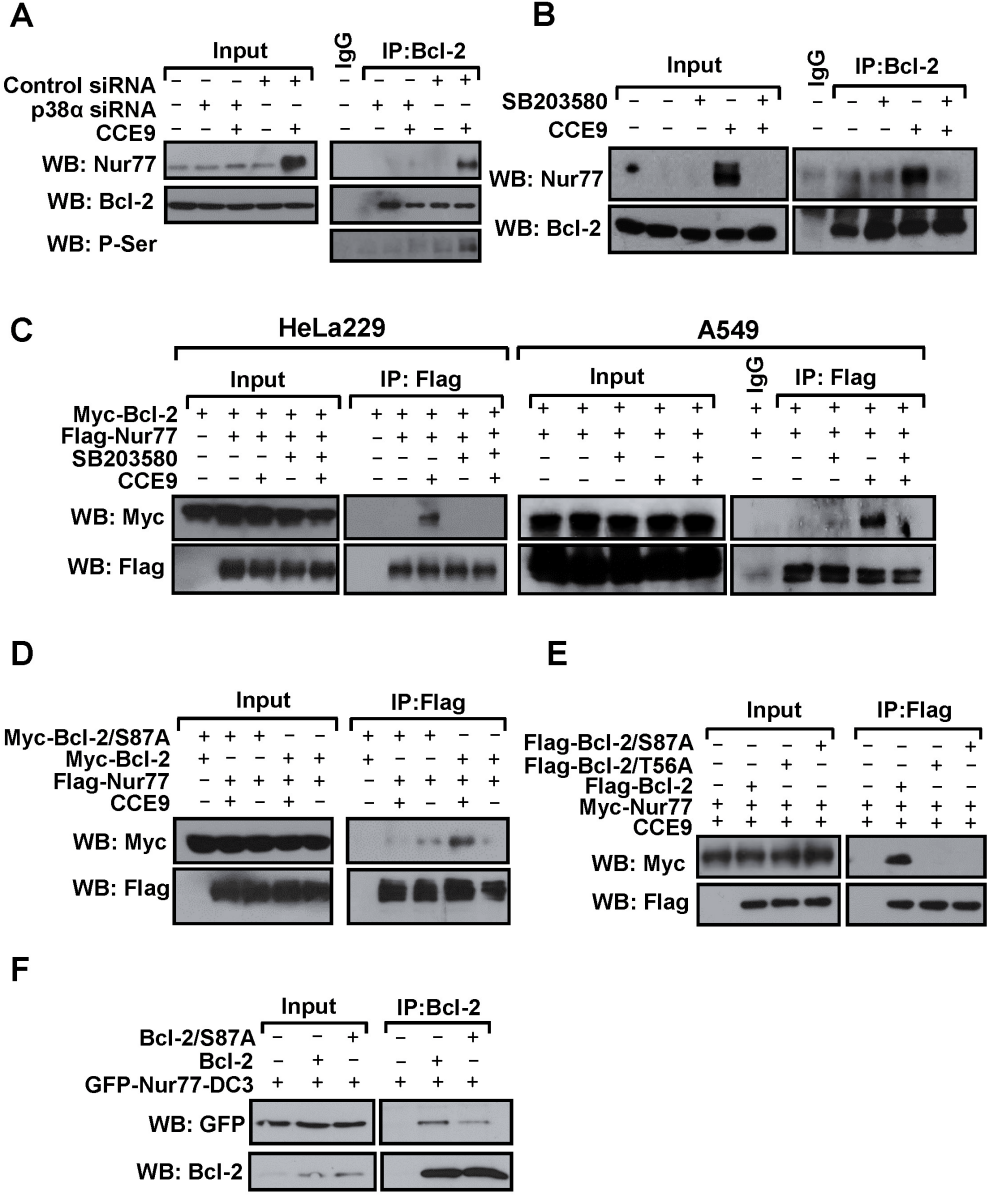
p38α MAPK regulate Nur77-Bcl-2 interaction **(A)** Transfection of p38α MAPK siRNA inhibits the induction of Nur77 interaction with Bcl-2 by CCE9. HeLa229 cells were transfected with p38α MAPK siRNA for 48 hr, treated with 10 μM CCE9 for 2 hr, and subjected to co-IP assays for Nur77 interaction with Bcl-2 by using anti-Bcl-2 antibody. Lysates and immunoprecipitates were examined by Western blotting using anti-Nur77, anti-Bcl-2, and anti-p-Ser antibodies. Input represents 10% of cell lysates used in the co-IP assays. **(B)** Effect of SB203580 on induction of Nur77 interaction with Bcl-2 by CCE9. HepG2 cells treated with 10 μM CCE9 for 3 hr in the presence or absence of 25 μM SB203580 were immunoprecipitated by using anti-Bcl-2 antibody. Lysates and immunoprecipitates were examined by Western blotting using anti-Nur77 and anti-Bcl-2 antibodies. Input represents 10% of cell lysates used in the co-IP assays. **(C)** Inhibitory effect of SB203580 on CCE9-induced interaction of transfected Nur77 and Bcl-2. Myc-Bcl-2 (2 μg) and Flag-Nur77 (2 μg) expression vector alone or together were transfected into HeLa229 or A549 cells. Cell lysates were immunoprecipitated by using anti-Flag antibody. Lysates and immunoprecipitates were examined by Western blotting using anti-Myc and anti-Flag antibodies. Input represents 5% of cell lysates used in the co-IP assays. **(D)** Myc-Bcl-2 (2 μg) or Myc-Bcl-2/S87A (2 μg) and Flag-Nur77 (2 μg) expression vector alone or together were transfected into HeLa229 cells. Cell lysates were immunoprecipitated by using anti-Flag antibody. Lysates and immunoprecipitates were examined by Western blotting using anti-Myc and anti-Flag antibodies. Input represents 5% of cell lysates used in the co-IP assays. **(E)** Flag-Nur77 (2 μg) was transfected alone or together with Flag-Bcl-2 (2 μg), Flag-Bcl-2/S87A (2 μg), or Flag-Bcl-2/T56A (2 μg) into Bcl-2-depeleted HeLa cells. Cell lysates were immunoprecipitated by using anti-Flag antibody. Lysates and immunoprecipitates were examined by Western blotting using anti-Myc and anti-Flag antibodies. Input represents 5% of cell lysates used in the co-IP assays. **(F)** Myc-Bcl-2 (2 μg) or Myc-Bcl-2/S87A (2 μg) and GFP-Nur77-DC3 (2 μg) expression vector alone or together were transfected into HEK293T cells. Cell lysates were immunoprecipitated by using anti-Bcl-2 antibody. Lysates and immunoprecipitates were examined by Western blotting using anti-GFP and anti-Bcl-2 antibodies. Input represents 5% of cell lysates used in the co-IP assays.

Bcl-2 phosphorylation by p38α MAPK is mainly mediated by multiple phosphorylation sites in its loop region. As the loop of Bcl-2 is responsible for Bcl-2 interaction with Nur77 and subsequently Bcl-2 conversion, we studied whether CCE9 activation of p38α MAPK was involved in the regulation of Bcl-2 interaction with Nur77. Co-immunoprecipitation assays demonstrated that Nur77 induced by CCE9 treatment was co-immunoprecipitated together with phosphorylated Bcl-2 by anti-Bcl-2 antibody (Figure 6A), demonstrating their interaction. Such an interaction was not observed in cells transfected with p38α MAPK siRNA (Figure 6A) or treated with SB203580 (Figure 6B), likely due to their inhibition of Nur77 expression and Bcl-2 phosphorylation. To exclude the effect of p38α MAPK inhibition on Nur77 expression, we studied the interaction of transfected Nur77 and Bcl-2. Thus, Myc-tagged Bcl-2 and Flag-tagged Nur77 were co-transfected into HeLa229 cells, and their interaction in the presence or absence of CCE9 was examined. Co-immunoprecipitation assays showed that transfected Flag-Nur77 and Myc-Bcl-2 strongly interacted when cells were treated with CCE9 in both HeLa229 and A549 cells (Figure 6C). However, when cells were co-treated with SB203580, which did not affect the expression of transfected Flag-Nur77, CCE9-induced Nur77-Bcl-2 interaction was strongly inhibited (Figure 6C). Thus, CCE9-induced Nur77 interaction with Bcl-2 likely involved its phosphorylation of Bcl-2 through its activation of p38α MAPK. To further characterize the role of Bcl-2 phosphorylation in its interaction with Nur77, Ser87 and T56 in the loop of Bcl-2, which was shown to be phosphorylated by p38α MAPK [23], were mutated into Ala. When Bcl-2/S87A, in which Ser87 was substituted with Ala, was analyzed for its interaction with Nur77 in HeLa229 cells, it failed to interact with Nur77 in cells treated with CCE9, while Bcl-2 strongly interacted with Nur77 under the same conditions (Figure 6D). CCE9 also showed much reduced effect on inducing Nur77 interaction with Bcl-2/S87A and another Bc-2 mutant with Thr56 substituted with Ala (Bcl-2/T56A) in Bcl-2-depleted HeLa cells, as compared to its inducing effect on Nur77 interaction with wild-type Bcl-2 (Figure 6E). Interaction of Nur77-DC3, a Nur77 mutant that constitutively resides in the cytoplasm to interact with Bcl-2 [7, 15], with Bcl-2/S87A was also reduced when compared to its binding to the wild-type Bcl-2 (Figure 6F). Together, these results demonstrate that p38α MAPK phosphorylation of Bcl-2 at Ser87 and Thr56 plays an important role in its interaction with Nur77.

## DISCUSSION

In studying the apoptotic effect of CCE9, we provided evidence here that it could induce apoptosis of various cancer cells through its activation of the Nur77-Bcl-2 apoptotic pathway. This is illustrated by our data showing that the apoptosis induction by CCE9 was accompanied with its induction of Nur77 expression (Figure 1B-1E), Nur77 mitochondrial targeting (Figure 3C), and Nur77 interaction with Bcl-2 (Figure 3E).

The Nur77-Bcl-2 apoptotic pathway involves the translocation of Nur77 from the nucleus to the cytoplasm, which was shown to be mediated by a CRM-1-dependent nuclear export process [5, 33, 34]. Previous studies demonstrated that JNK activation was required for Nur77 nuclear export by certain apoptotic stimuli such as 3-Cl-AHPC [9, 11, 32, 33]. Our current study however showed that JNK was only moderately activated by CCE9 (Figure 4D). In addition, treatment of cells with LMB failed to block CCE9-induced Nur77 cytoplasmic accumulation (Figure 4A) and apoptosis induction (Figure 4B-4C). Furthermore, Bcl-2 expression was required for CCE9-induced cytoplasmic accumulation of Nur77 protein (Figure 3D-3E). These observations together with our results that CCE9 could induce Nur77 interaction with cytoplasmic Bcl-2 (Figure 6) demonstrated that CCE9-induced cytoplasmic localization of Nur77 is likely due to the cytoplasmic retention of the Nur77 protein by Bcl-2 through protein-protein interaction. Our data that Bcl-2 mutants capable of binding Nur77 could retain Nur77 in the cytoplasm while Bcl-2 mutant defective in Nur77 binding could not (Figure 3F) support this conclusion. Thus, CCE9 may employ a different mechanism to activate the Nur77-Bcl-2 pathway.

Indeed, we provide evidence that activation of p38α MAPK by CCE9 plays a critical role in the regulation of the Nur77-Bcl-2 apoptotic pathway. The apoptotic effect of p38α MAPK involving its modulation of death receptors, survival pathways, or pro- and anti-apoptotic Bcl-2 proteins has been well described [21, 22]. Diverse chemotherapeutic agents, including microtubule-perturbing drugs vinblastine, vincristine and paclitaxel and DNA damaging agent cisplatin, could stimulate apoptosis in a p38α MAPK-dependent manner [21, 22]. Our results showed that CCE9 markedly activated p38α MAPK in HeLa229 cells (Figure 4D), which coincided with its induction of Nur77 expression and apoptosis (Figure 4E, 5A-5B). Treatment of cells with the p38α inhibitor (SB203580) or knocking down p38α MAPK potently inhibited the ability of CCE9 to induce PARP cleavage (Figure 4E, 4H), caspase-3 activation (Figure 4F), and Bcl-2 conformational change (Figure 5F-5G) in HeLa229 Cells. Induction of PARP cleavage by CCE9 was also impaired in p38α MAPK Knockout cells (Figure 5D). These data reveal a critical role of p38α MAPK activation in mediating the apoptotic effects of CCE9.

The activation of the Nur77-Bcl-2 apoptotic pathway by CCE9 involves its induction of Nur77. Inhibition of p38α MAPK activation by SB203580 (Figure 5A) or transfection of p38α MAPK siRNA (Figure 5B) could inhibit the ability of CCE9 to induce Nur77 expression. Moreover, the level of Nur77 protein was dramatically reduced in p38α^-/-^MEFs as compared to the wild-type MEFs (Figure 5D). How p38α MAPK acts to mediate the induction of Nur77 by CCE9 remains to be studied. It has been shown that p38α MAPK activation by CD437, a Nur77 inducer, could phosphorylate MEF2 that serves as a transcriptional regulator of Nur77 [9]. Another study showed that induction of Nur77 by p38α MAPK requires its activation of mitogen-and stress-activated protein kinase (MSK), which in turn phosphorylates CREB known to bind to two AP-1-like elements present in the Nur77 promoter [35]. However, our RT-PCR data (Figure 1F) demonstrated that CCE9 induction of Nur77 expression is unlikely mediated by its transcriptional regulation.

Our results revealed an important role of p38α MAPK activation in promoting the interaction between Nur77 and Bcl-2. Cytoplasmic Nur77 is known to interact with Bcl-2, inducing a Bcl-2 conformational change that is pro-apoptotic [7]. CCE9-induced interaction of Nur77 with Bcl-2 was inhibited by transfection of p38α MAPK siRNA (Figure 6A) or treatment with SB203580 (Figure 6B, 6C), demonstrating that CCE9 induction of the interaction of Nur77 with Bcl-2 depended on its activation of p38α MAPK. Our results are in agreement with a previous report showing that inhibition of p38α MAPK blocked the effect of α-lipoic acid on inducing apoptosis and Nur77 cytoplasmic localization in vascular smooth muscle cells [11]. Binding of Bcl-2 to Nur77 is mediated by its natively unstructured loop region [7, 15]. Phosphorylation in the loop region is known to suppress the anti-apoptotic function of Bcl-2 likely through its interference with Bcl-2 interaction with pro-apoptotic Bcl-2 family proteins. Bcl-2 is phosphorylated by p38α MAPK upon NGF withdrawal in memory B lymphocytes, leading to inhibition of its anti-apoptotic activity and induction of cytochrome *c* release [24]. Similarly, H_2_O_2_ induces p38α MAPK-mediated Bcl-2 phosphorylation in adult rat cardiac myocytes [36], contributing to apoptosis [37]. Ser87 and Thr56 residues located in the unstructured loop region of Bcl-2 have been shown to be phosphorylated by p38α MAPK and the phosphorylation of these residues is associated with a decrease in the anti-apoptotic potential of Bcl-2 protein [23]. Our findings that p38α MAPK activation by CCE9 resulted in Bcl-2 phosphorylation (Figure 5E) and that mutating the p38α phosphorylation sites in the loop of Bcl-2 reduced its interaction with Nur77 (Figure 6D-6F) reveal a critical role of p38α MAPK phosphorylation of Bcl-2 in the regulation of Bcl-2 interaction with Nur77, an event important for apoptosis induction. Thus, our results also provide new insight into the mechanism by which p38α MAPK mediates the apoptotic effects of diverse chemotherapeutic agents.

## MATERIALS AND METHODS

### Reagents

Lipofectamine 2000 from Invitrogen (Carlsbad, CA, USA); goat anti-rabbit and anti-mouse secondary antibody conjugated to horseradish peroxidase from Thermo Fisher Scientific, Inc. (Waltham, MA, USA); anti-mouse/rabbit IgG conjugated with Cy3, anti-mouse/rabbit IgG conjugated with FITC from Chemicon International (Temecula, CA, USA); anti-Nur77 (3960), anti-P-p38 (9215), anti-p38 (9212), anti-P-JNK (9251), anti-JNK (9252), anti–cleaved caspase-3 (9664), and anti-Tubulin (2144) antibodies from Cell Signal Technology; anti–Bcl-2 (sc-509), anti-Bax (6A7; sc-23959), and anti-Myc (9E10; sc-40) antibodies from Santa Cruz Biotechnology (Santa Cruz, CA, USA); anti–poly (ADP-ribose) polymerase antibody (PARP; 556494) from BD Biosciences (San Diego, CA, USA); anti-β-actin, anti-Flag (M2), anti-P-Ser (p3430), and anti-P-Thr (p3555) antibodies from Sigma (St. Louis, MO, USA); SB203580, SP600125, leptomycin B (LMB), from Sigma; anti-Bcl-2 BH3 (AP1303a) from Abgent (San Diego, CA, USA); polyvinylidene difluoride membranes from Millipore (Billerica, MA, USA); and enhanced chemiluminescence reagents from GE Healthcare (Buckinghamshire, UK) and a cocktail of proteinase inhibitors from Roche (Meylan, France) were used in this study. All other chemicals used were commercial products of analytic grade obtained from Sigma (St. Louis, MO, USA).

### Cell culture and transient transfection

HeLa cells, HeLa229 cells and HeLa Bcl-2 knockout cells were cultured in Eagle's Minimum Essential Medium containing 10% fetal bovine serum (FBS), HepG2 cells, A549 cells, HEK293T cells, MEFs, p38α MAPK^-/-^MEFs and Bcl-2^-/-^MEFs were cultured in Dulbecco’s modified Eagle’s medium containing 10% fetal bovine serum (FBS). Subconfluent cells with exponential growth were used throughout the experiments. Transient transfection was performed using Lipofectamine 2000 (Invitrogen) according to the instructions of the manufacturer in regular growth medium.

### Plasmid constructions

Expression vectors for Nur77, Bcl-2, Bcl-2/Δ TM, Bcl-2/Δ BH4, Bcl-2/Δ Loop and Bcl-2/S87A mutants were described previously [7, 15]. Bcl-2/T56A mutant was generated by Fast Site-Directed Mutagenesis Kit from TIANGEN (Beijing, China).

### siRNA and transfections

siRNAs against Nur77 (SASI_Hs02_00333289, SASI_Hs02_00333290, SASI_Hs02_00333291), p38α MAPK (SASI_Hs02_00331604, SASI_Hs01_00018466, SASI_Hs02_00331605), and Bcl-2 (SASI_Hs01_00119086, SASI_Hs01_001190867, SASI_Hs01_001190868) and control siRNA used were from Sigma. 5 μL aliquot of 20 μM siRNA/well was transfected into cells in six-well plates using Lipofectamine 2000 (Invitrogen), according to the manufacturer’s recommendations.

### Cell lysis and fractionation

Cell lysates were prepared by lysing cells with modified radioimmunoprecipitation assay buffer containing 50 mM Tris-HCl (pH 7.4), 150 mM NaCl, 5 mM EDTA, 1% NP40, 0.5% sodium deoxycholate, and 0.1% SDS with a cocktail of proteinase inhibitors on ice for 30 min. For cellular fractionation, cells were lysed in cold buffer [10 mM HEPES-KOH (pH 7.9), 1.5 mM MgCl_2_, 10 mM KCl, and 0.5 mM DTT] with a cocktail of proteinase inhibitors on ice for 10 min. The cytoplasmic fraction was collected by centrifuging at 6,000 rpm for 30 sec. Pellets containing nuclei were resuspended in cold high-salt buffer [20 mM HEPES-KOH (pH 7.9), 25% glycerol, 420 mM NaCl, 1.5 mM MgCl_2_, 0.2 mM EDTA, 0.5 mM DTT], with a cocktail of proteinase inhibitors on ice for 30 min. Cellular debris was removed by centrifugation at 12,000 rpm at 4°C for 15 min.

### Western blotting

Equal amounts of the lysates were electrophoresed on an SDS-PAGE gel (8 or 12%) and transferred onto polyvinylidene difluoride membranes, which were then blocked with 5% nonfat milk in TBST [50 mM Tris-HCl (pH 7.4), 150 mM NaCl, and 0.1% Tween 20] for 1 hr, incubated with various primary antibodies for 2 hr at room temperature and incubated with secondary antibodies for 1 hr. Immunoreactive products were detected by chemiluminescence with an enhanced chemiluminescence system (GE Healthcare).

### Co-immunoprecipitation assays

For co-immunoprecipitation (co-IP) assay [7, 15, 38], cell lysates were incubated with the appropriate antibody for 1 hr, and subsequently incubated with protein A-Sepharose beads for 1 hr. The protein–antibody complexes recovered on beads were subjected to western blotting using appropriate antibodies after separation by SDS-polyacrylamide gel electrophoresis.

### Immunofluorescence microscopy

Cells mounted on glass slides were permeabilized with PBS containing 0.1% Triton X-100 for 15 min, and blocked with 1% bovine serum albumin (BSA) in PBS for 30 min at room temperature, followed with incubation with various primary antibodies at 37°C for 1 hr and detected by FITC-labeled anti-mouse/rabbit IgG (Chemicon International) or anti-mouse/rabbit IgG conjugated with Cy3 (Chemicon International) at room temperature for 30 min. Cells were co-stained with 4’6’-diamidino-2-phenylindole (DAPI) (Sigma) to visualize nuclei. The images were taken under an LSM-510 confocal laser scanning microscope system (Carl Zeiss, Oberkochen, Germany).

### Apoptosis assays

For DAPI staining, cells were collected and incubated in PBS containing 50 μg/mL of DAPI and 100 μg/mL of DNase-free RNase A at 37°C for 20 min. Apoptotic cells were identified with typical morphology of shrinkage of the cytoplasm, membrane blebbing, and nuclear condensation and/or fragmentation. At least 300 cells from more than five random microscopic fields were counted by two independent investigators.

### Reverse transcription-PCR analysis

Total RNAs were isolated by Trizol LS. The first-strand synthesis was performed with Revert Aid First-Strand cDNA synthesis kits (Fermentas) according to the instructions of the manufacturer. The primers include those for Nur77 (forward primer, 5’-TCA TGG ACG GCT ACA CAG-3’, reverse primer, 5’-GTA GGC ATG GAA TAG CTC-3’) and β-actin (forward primer, 5’-CTG GAG AAG AGC TAC GAG-3’, reverse primer, 5’-TGA TGG AGT TGA AGG TAG-3’). PCR reactions were performed in Eppendorf AG 22331 Hamburg (Eppendorf), and analyzed by electrophoresis using 2% agarose gels. Gel images were captured with a Gel logic 200 system (Kodak).

### Flow cytometric analysis

Apoptosis was determined by dual staining using Annexin V/FITC and propidium iodide (Invitrogen). Briefly, cells were seeded into 24-well cell culture plates (1×10^5^ cells/well) and treated with CCE9 for 6 hr. Cells were dissociated from wells with 0.25% trypsin, spun at 1,500 rpm for 5 min, resuspended in Annexin V binding buffer, and stained with 1 μL Annexin V/FITC for 15 min and 1 μL propidium iodide for 1 min. Cells were analyzed using the FACSCalibur System (BD Biosciences, San Jose, CA, USA). The relative proportion of Annexin V-positive cells, representing apoptotic cells, was determined using FlowJo software (FlowJo LLC, Ashland, OR, USA).

### Statistical analysis

Data were expressed as mean±SD. Each assay was repeated in triplicate in three independent experiments. Statistical significance of differences between groups was analyzed by using Student’s t test or ANOVA analysis. p<0.05 was considered significant.

## Abbreviations

MAPK: mitogen-activated protein kinase
PARP: poly ADP-ribose polymerase
siRNA: small interfering RNA
BH domain: Bcl-2 homology domain
JNK: c-Jun N-terminal kinase
DAPI: 4’6’-diamidino-2-phenylindole
LMB: leptomycin B
H_2_O_2_: hydrogen peroxide
GFP: green fluorescent protein
